# Problem solving therapy (PST) tailored for intimate partner violence (IPV) versus standard PST and enhanced usual care for pregnant women experiencing IPV in rural Ethiopia: protocol for a randomised controlled feasibility trial

**DOI:** 10.1186/s13063-020-04331-0

**Published:** 2020-06-01

**Authors:** Roxanne C. Keynejad, Tesera Bitew, Katherine Sorsdahl, Bronwyn Myers, Simone Honikman, Girmay Medhin, Negussie Deyessa, Nick Sevdalis, Wietse A. Tol, Louise Howard, Charlotte Hanlon

**Affiliations:** 1 Section of Women’s Mental Health, Health Service and Population Research Department, Institute of Psychiatry, Psychology & Neuroscience, King’s College London, London, UK; 2 College of Health Sciences, Addis Ababa University, Addis Ababa, Ethiopia; 3 Institute of Educational and Behavioural Science, Debre Markos University, Debre Markos, Ethiopia; 4 Alan J. Flisher Centre for Public Mental Health, Department of Psychiatry and Mental Health, University of Cape Town, Cape Town, South Africa; 5 Alcohol Tobacco and Other Drug Use Research Unit, South African Medical Research Council, Cape Town, South Africa; 6 Department of Psychiatry and Mental Health, University of Cape Town, Cape Town, South Africa; 7 Perinatal Mental Health Project, Alan J. Flisher Centre for Public Mental Health, Department of Psychiatry and Mental Health, University of Cape Town, Cape Town, South Africa; 8 Aklilu-Lemma Institute of Pathobiology, Addis Ababa University, Addis Ababa, Ethiopia; 9 College of Health Sciences, Addis Ababa University, Addis Ababa, Ethiopia; 10 Centre for Implementation Science, Health Service and Population Research Department, Institute of Psychiatry, Psychology & Neuroscience, King’s College London, London, UK; 11 Department of Mental Health, Bloomberg School of Public Health, Johns Hopkins University, Baltimore, MD USA; 12 United States of America (USA) & Peter C. Alderman Program for Global Mental Health, HealthRight International, New York, New York, NY USA; 13 Centre for Global Mental Health, Health Service and Population Research Department, Institute of Psychiatry, Psychology & Neuroscience, King’s College London, London, UK; 14 World Health Organization Collaborating Centre for Mental Health Research and Capacity-Building, Department of Psychiatry, School of Medicine, College of Health Sciences, Addis Ababa University, Addis Ababa, Ethiopia; 15 Centre for Innovative Drug Development and Therapeutic Trials for Africa (CDT-Africa), College of Health Sciences, Addis Ababa University, Addis Ababa, Ethiopia

**Keywords:** Global mental health, Pregnancy, Perinatal mental health, Intimate partner violence, Psychological interventions, Task sharing, Low- and middle-income countries, Feasibility studies, Implementation research

## Abstract

**Background:**

In rural Ethiopia, 72% of women are exposed to lifetime intimate partner violence (IPV); IPV is most prevalent during pregnancy. As well as adversely affecting women’s physical and mental health, IPV also increases the risk of child morbidity and mortality associated with maternal depression, thus making antenatal care an important opportunity for intervention. Adapting generic, task-shared, brief psychological interventions for perinatal depression and anxiety to address the needs and experiences of women affected by IPV may improve acceptability to women and feasibility for health workers. This randomised controlled feasibility trial will compare brief problem solving therapy (PST) specifically adapted for pregnant women experiencing IPV (PST-IPV) with standard PST and enhanced usual care to determine the feasibility of a future fully powered randomised controlled trial.

**Methods:**

Seventy-five pregnant women scoring five or more on the Patient Health Questionnaire, endorsing a tenth question about functional impact and reporting past-year IPV, will be recruited from antenatal care clinics in predominantly rural districts in Ethiopia. Consenting participants will be randomised to either four sessions of PST-IPV, four sessions of standard PST or information about sources of support (enhanced usual care) in a three-arm design. The interventions will be delivered by trained, supervised antenatal care staff using a task-sharing model. Assessments will be made at baseline and after 9 weeks by masked outcome assessors and will include measures of depression symptoms (primary outcome), post-traumatic stress, anxiety symptoms, functional impact, past-month IPV and hypothesised mediators (secondary outcomes). A mixed-method process evaluation will determine the feasibility of a future randomised controlled trial, assess the feasibility, acceptability, fidelity and quality of implementation of PST-IPV, generate testable hypotheses about causal mechanisms, and identify potential contextual factors influencing outcomes.

**Discussion:**

Despite mental health being a critical concern for women experiencing IPV, there is limited evidence for brief, task-shared psychological interventions adapted for their needs in low- and middle-income countries. Contextually tailored interventions for pregnant women experiencing IPV in low- and middle-income countries require development and process evaluation. This randomised controlled feasibility trial will yield results on the feasibility of conducting a fully powered trial, relevant to researchers, primary and antenatal care clinicians in resource-limited settings.

**Trial registration:**

Pan-African clinical trials registry: PACTR202002513482084. Prospectively registered on 13 December 2019.

## Introduction

### Background and rationale

Intimate partner violence (IPV) refers to behaviour by a partner or ex-partner that causes (or has the potential to cause) physical, sexual or psychological harm. IPV includes physical aggression, sexual coercion, psychological abuse and controlling activity [1]. It is highly prevalent worldwide [2] and an important social determinant of physical and mental health [3, 4]. In response, the World Health Organization (WHO) [5], the United Nations [6] and the World Psychiatric Association [7–9] have prioritised interventions to prevent and address IPV and its health impacts.

The relationship between IPV and mental health is bidirectional, such that IPV increases a woman’s risk of mental disorders, which in turn increases a woman’s vulnerability to (further) IPV. For example, IPV is associated with subsequent depressive symptoms, suicide attempts [10] and alcohol use disorders [11], which increase women’s risk for IPV (re)victimisation. Exposure to IPV is rarely assessed in randomised controlled trials (RCTs) of psychological interventions [12], but some studies have found reduced IPV alongside improved mental health [13] and birth outcomes [14].

There is limited evidence on mental health interventions tailored to the needs of pregnant women experiencing IPV [15], especially in low- and middle-income countries (LMICs). In a rapid review of evidence, 33 studies were identified that reported mental health interventions for women affected by IPV [16]. In most of these studies, the interventions were group-delivered cognitive behavioural therapy (CBT)-informed interventions, mind–body interventions (such as mindfulness-based stress reduction, yogic techniques and biofeedback) or individually delivered, trauma-focused psychotherapeutic interventions. Although all the identified studies came from high-income countries, protocols from Tanzania [17] and South Africa [18] indicated growing research interest in the African region. For example, one RCT in Nairobi, Kenya, has shown that five sessions of the multicomponent behavioural treatment Problem Management Plus (PM+) delivered to women with a history of any gender-based violence are associated with improved psychological distress and post-traumatic stress symptoms at 3-month follow-up compared with enhanced usual care [19].

A recent systematic review confirmed the paucity of intervention research addressing the mental health of both survivors and perpetrators of IPV in LMICs [20]; no studies replicated evaluations of previously studied interventions and none were conducted in low-income countries. The authors recommended strengthening the theoretical underpinnings of mental health interventions for people experiencing or perpetrating IPV, testing their impacts on hypothesised mediators and improving IPV detection using continuous outcome measures and fully powered samples. Treating depression and post-traumatic stress disorder (PTSD) has the potential to reduce self-blame, low self-esteem, hopelessness and emotional numbing, and improve communication, stress coping and anger management skills among both survivors and perpetrators [20].

We recently investigated whether IPV exposure moderates the efficacy of generic psychological interventions in LMICs. Our meta-analysis of 15 studies that provided data showed that women reporting IPV demonstrated greater improvements in anxiety symptoms than women not reporting IPV following generic psychological interventions (difference in standardised mean differences, 0.31; 95% confidence interval 0.04–0.57; *I*^2^ 49.3%) and consistent but non-significant differences in PTSD, depression and psychological distress symptom improvements [21]. Our systematic review only identified two RCTs of psychological interventions for depression or anxiety in LMICs which were tailored for women experiencing IPV. In Pakistan, ten sessions of IPV-adapted group CBT were associated with improved depression and anxiety compared with CBT-based self-help groups [22]. In Iran, 10–12 sessions of IPV-tailored narrative exposure therapy were associated with improved PTSD and depression at 3- and 6-month follow-up compared with treatment as usual (life skills training and supportive counselling) [23]. These studies support the potential benefits of adapting psychological interventions for depression, anxiety or PTSD to meet the needs and experiences of women affected by IPV.

Although PM+ has been shown to be effective for women experiencing gender-based violence in Nairobi [19], delivering five 90-min sessions may not be feasible in primary care settings in low-income countries. Problem solving therapy (PST) is a brief psychological intervention that aims to improve coping with life problems by teaching problem solving skills. A meta-analysis of PST for depression found a standardised mean effect size of 0.34, but high heterogeneity among included studies indicated the need for research to determine the settings and patient groups for whom PST is most effective [24]. A meta-analysis of PST for any mental or physical health problem found that it was significantly more effective than no treatment, treatment as usual, and ‘attention placebo’ arms (controlling for non-specific effects of contact), moderated by the use of problem orientation training and homework assignments [25].

Several studies from LMICs suggest that PST can be effective for treating depression, anxiety and psychological distress. A 5-week pilot study of adapted PST in English, Xhosa and Afrikaans in South African township residents found that it was acceptable, feasible and associated with significant reductions in psychological distress [26]. An RCT of South African emergency department attendees found that substance use was significantly reduced at 3-month follow-up in participants who received five sessions of blended PST and motivational interviewing, compared to motivational interviewing alone or psychoeducation control [27]. In Zimbabwe, the ‘Friendship Bench’ intervention comprised six sessions of individual PST delivered by lay health workers, and an optional six-session peer support group [28]. In this 86% female sample, depression and psychological distress were significantly reduced following PST in comparison to enhanced usual care.

The importance of designing RCTs to evaluate mediators of treatment and mechanisms of change is widely supported [29]. However, despite evidence of efficacy, studies of how PST works are limited and contradictory. Hypothesised mechanisms have included improving mastery, self-control and the accuracy of perceived problem severity [30], problem solving skills [31], ‘life integration’ [32] and locus of control (the extent to which the individual attributes their experiences to internal or external factors) [33]. An RCT comparing online CBT, PST and waiting list control for depression found that the effects of both interventions were mediated by reduced dysfunctional attitudes, worry, negativity towards problems and increased mastery, with no difference in effect sizes between CBT and PST [34]. The authors postulated that both interventions increase expectations of self-efficacy, leading to greater commencement and continuation of coping behaviours [35]. Both mechanisms for improved mood might also influence the ability of women to respond to IPV.

Process evaluations of complex intervention feasibility studies are increasingly recognised as being vital to optimise the safety, efficiency and validity of subsequent RCTs [5, 36]. Updated guidance [37] and growing consensus [38] support the need for studies to determine the feasibility of research methods used to study complex interventions before conducting definitive RCTs. Sociocultural, health system and economic factors affect the adaptation, translation, mechanisms, success and scale-up of interventions, so a mixed-method process evaluation [39] is crucial and helps to inform understanding of the context for future implementation [40].

In rural Ethiopia, 72% of women are exposed to lifetime IPV [41] and this is associated with emotional distress [42] and depression [43]. IPV in Ethiopia is most prevalent during pregnancy [44] and increases the risk of child morbidity and mortality associated with maternal depression [45]. Antenatal depression in Ethiopia is associated with increased emergency presentations in pregnancy [46], perinatal complications [47], prolonged labour [48] and use of emergency delivery care [49]. As pregnancy is the most common time for Ethiopian women to access health care [50], antenatal care offers an important opportunity to provide an intervention that addresses both IPV and depression.

The range of cultural, geographical, economic, linguistic, religious, socio-political, health system and other differences between rural Ethiopia and the largely middle-income settings of most RCTs published to date supports the need for research evaluating PST adapted for women experiencing IPV (PST-IPV) in this context.

### Objectives

In this randomised feasibility trial and process evaluation of perinatal PST-IPV in rural Ethiopia, we aim to determine the feasibility and acceptability of the intervention and study design to inform a future fully powered RCT. Specific objectives are to:

1. Determine whether the PST-IPV intervention and processes of recruitment, randomisation, follow-up and evaluation are feasible and acceptable to pregnant women and health workers in rural Ethiopia. This includes estimating parameters to inform the design of a future RCT.
2. Refine the PST-IPV intervention and study design in response to a mixed-method process evaluation, which assesses the feasibility, acceptability, fidelity and quality of the intervention, explores causal mechanisms, and identifies contextual factors that may influence outcomes [37].

### Trial design

In this three-arm feasibility trial, we will randomise eligible women (with depressive symptoms, functional impact and past-year IPV) to PST-IPV, standard PST or enhanced usual care (information about relevant sources of support). All arms represent additions to standard clinical care, which does not currently provide any routine interventions for perinatal depression or IPV.

**Fig. 1:**
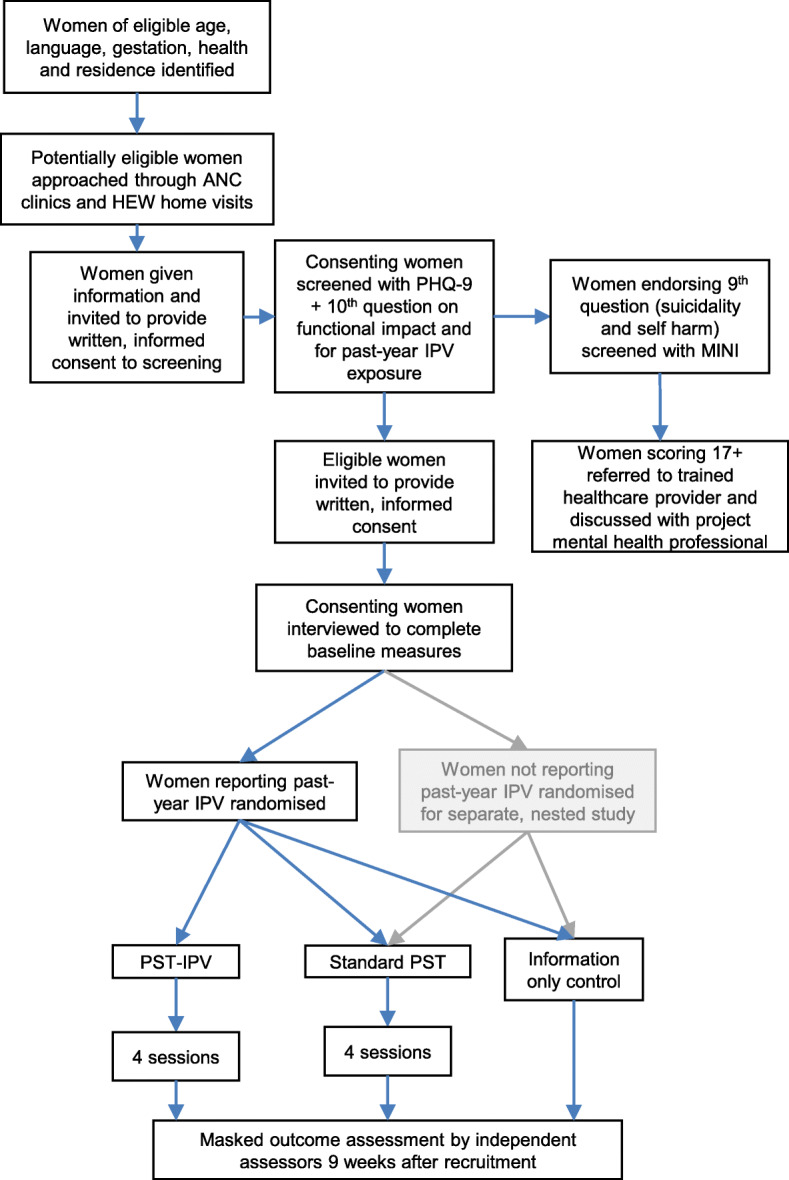
Flow diagram of problem solving therapy (PST) adapted for pregnant women experiencing intimate partner violence (IPV) feasibility trial procedure, indicating the relationship with a nested feasibility trial of standard PST in women not reporting past-year IPV (shaded grey). ANC antenatal care, HEW health extension worker, MINI Mini-International Neuropsychiatric Interview, PHQ-9 Nine-item Patient Health Questionnaire

This study is linked to a two-arm feasibility trial that will randomise eligible women (with depressive symptoms and functional impact) to standard PST or information only, the protocol of which is reported separately (Bitew T, Keynejad RC, Honikman S, Sorsdahl K, Myers B, Fekadu A, et al. Brief problem-solving therapy for perinatal depression in rural Ethiopia: protocol for a randomised feasibility study.) (see Fig. 1).

## Methods

### Study setting

The study will be conducted in selected health facilities in the predominantly rural areas of Gurage zone (projected population 1,712,506) and Silt’e zone (projected population 1,043,242) in the Southern Nations, Nationalities, and Peoples’ Region of Ethiopia [51]. Primary health care is structured so that the nurse, midwife and health officer-run health centres (serving a population of 25,000–40,000) are linked to five community health posts (serving a population of 3000–5000) staffed by one or two health extension workers (HEWs) each, with access to a single primary hospital for more specialist care. HEWs, who have completed high school and received 1 year of undergraduate-level training [52], provide a first antenatal contact before referring women for further antenatal care (ANC) at a health centre or primary hospital and maintain contact with women during pregnancy.

This study builds on the programme for improving mental health care (PRIME) study [53], which integrated mental health into primary and maternal care in five LMICs, including the Gurage zone, Ethiopia [54]. In PRIME, a district-level mental health plan was developed in collaboration with key stakeholders and comprised interventions at the community, health facility and health system level. At the facility level, PRIME trained primary and maternal care staff using the mental health gap action programme (mhGAP) intervention guide of the WHO, which guides non-mental health specialists through clinical diagnostic and treatment algorithms for prioritised mental, neurological and substance use disorders [55]. The PRIME study investigators identified low detection rates of depression, which may be related in part to a lack of acceptable treatment options. Formative work was therefore conducted to inform adaptation of a brief psychological intervention for depression. In a nested study, PST was then adapted for antenatal women in this rural Ethiopian setting.

A systematic review and meta-analysis [21], further qualitative interviews and theory of change workshops (Bitew T, Keynejad RC, Honikman S, Sorsdahl K, Myers B, Fekadu A, et al. Brief psychological intervention for antenatal depression: a qualitative study.) (Keynejad RC, Bitew T, Mulushoa A, Tol W, Howard LM, Hanlon C. Adapting brief, task-shared problem-solving therapy for women experiencing intimate partner violence in rural Ethiopia: a qualitative study.) were then used to adapt this locally tailored PST intervention to address the needs and experiences of women affected by IPV. The preliminary theory of change visualises health worker perspectives on the components, hypothesised mediators, intermediate and long-term outcomes of PST-IPV intervention, and factors anticipated to influence the delivery and implementation of PST-IPV (see Supplementary file 1).

Adaptation for IPV focused on international guidelines [5], which recommend identifying women experiencing IPV, training health care providers about IPV, and providing woman-centred care and support when IPV is disclosed. PST-IPV will apply a PST approach in the context of IPV, enabling IPV-related problem solving and management of mental health-related symptoms.

### Participant sample size

As a feasibility trial, this study is not powered to detect intervention efficacy, but rather to estimate feasibility parameters to inform a future RCT, and test intervention and research protocols. With a total sample size of 75 (25 participants randomised to PST-IPV, 25 to PST and 25 to enhanced usual care), a drop-out rate of 30% can be estimated to within a 95% confidence interval of ±3%. To estimate the standard deviation of the primary feasibility outcome measure to inform a future RCT sample size calculation, recommended feasibility trial sample sizes range between a total of 24 and 50 across two arms [56, 57].

### Participant recruitment

Research staff will consult health care workers based in primary hospitals and/or health centres in Gurage and Silt’e zones to identify potentially eligible women meeting those inclusion criteria about which information is held for screening. Potentially eligible women will be approached through ANC clinics and HEW home visits by research staff and provided with written and verbal information in Amharic, before being invited to give written, informed consent to initial screening. Non-literate women will signify their consent by finger print. A high school-educated assistant will act as a witness to confirm that the information sheet has been read aloud correctly to non-literate women. Women screened as eligible to participate in the randomised feasibility trial will then be invited to give written, informed consent to participate. As with screening, non-literate women will signify their consent to participate in the trial by witnessed finger print.

Due to potential risks of abusive partners learning of women’s involvement, information sheets, consent forms and other study paperwork will be kept in the locked research office after being read and signed but will be accessible to women at their request. Due to logistical challenges affecting women’s daily lives in this setting, it is not possible to give prospective participants 24 h to decide whether to take part. However, a minimum of 30 min will be allowed for the woman to make up her mind about participating in the study.

### Participant screening

Consecutive, potentially eligible women who provide informed consent will be screened for depressive symptoms and a history of past-year IPV after their ANC appointment. Depressive symptoms will be screened for using the locally validated [58] nine-item Patient Health Questionnaire (PHQ-9) [59]. To screen for IPV exposure, research staff will first read the introductory paragraph of the Amharic translated [60] Conflict Tactics Scale [61] to potential participants (or they may read themselves, if literate) to ameliorate stigma they may feel towards disclosing IPV. A five-item ‘non-graphic language’ screening test previously used in this and other LMIC contexts and found to be a valid measure of IPV [62] will then be administered. Finally, items from the WHO multi-country study [42] of women’s health and domestic violence, previously used in this setting, will be used to ask about experiences of IPV in the past year.

All screened women scoring five or more on the PHQ-9 [63, 64] and endorsing any functional impact of symptoms (the tenth item) will be invited to participate in the research study. Endorsement of the tenth item will be defined as answering “somewhat”, “very” or “extremely difficult” to the question “over the last two weeks, how difficult have these problems made it for you to do your work, take care of things at home, or get along with other people?”.

Consenting women who disclose any past-year IPV during screening will be randomised to one of PST-IPV, standard PST or enhanced usual care, whereas consenting women reporting no IPV on baseline measurements will be randomised to one of standard PST or enhanced usual care for the separate, nested PST feasibility trial (see Fig. 1).

### Participant recruitment procedures

All women screened as eligible to participate will be informed about the study and given the opportunity to ask questions before being invited to consent to participate in the randomised feasibility trial. Women consenting to participate will first receive their routine clinical care and then participate in a baseline interview using fully structured questionnaires. If screening or this assessment identify any sources of concern (e.g. suicidal ideation, risks to herself or others), the researcher will discuss these with the woman and explain the need to share them with her health care professional, before involving them. To be eligible to participate in the PST-IPV components of the overall study, women must meet the eligibility criteria (see below).

### Participant eligibility criteria

Women can be included if they:

- Speak Amharic (the official regional language)
- Are aged 16 years or over
- Are between 12 and 34 weeks gestation of pregnancy
- Are intending to reside in the study area for the duration of the study
- Score 5 or more on PHQ-9 with functional impairment (tenth question)
- Report IPV in the past year (in a current or previous relationship) during screening
- Consent to participate, including to accept enhanced usual care or to attend four sessions of PST-IPV or PST (if randomised to a treatment arm)

Women are required to speak Amharic, the official language of the region, so that they can access all translated study materials, verbally or in writing. Relationships are defined as any romantic or sexual interaction within or outside marriage.

PHQ-9 has been validated in the study area in both primary care [63] and ANC attendees (area under the receiver operating characteristic curve 0.91, 95% confidence interval 0.86–0.96) [64]. For primary care attendees a score of five or more was the optimal cut-off for identifying possible depressive disorder. For antenatal women, a cut-off of four or more was optimal. However, since at the optimal cut-offs the positive predictive value was less than 50%, in this study women scoring above the cut-off (five or more) will be included if they also report difficulty in their day-to-day activities (measured using the PHQ-9 tenth item).

### Participant exclusion criteria

Women will be excluded if they:

- Are acutely unwell
- Require emergency treatment
- Are identified by the ANC provider during pre-screening as having possible psychotic symptoms
- Are unable to understand the interview (e.g. diagnosed with severe intellectual disability or dementia, or unable to speak Amharic)
- Expect to move away from the study area before the study is completed

All women endorsing question nine of PHQ-9 about suicidal thoughts or self-harm behaviours will receive the Amharic [63] Mini-International Neuropsychiatric Interview suicidality scale [65]. Women scoring 17 or above on the Mini-International Neuropsychiatric Interview (indicating high suicide risk) will be excluded from participation as part of a stepped-care model [54]. They will be referred instead to a mental health-trained primary care worker who will employ mhGAP [55] criteria to assess for imminent risk of self-harm or suicide. Women assessed as being at imminent risk will be referred to a psychiatric nurse in the primary hospital for escalation of their care. The attendance of participants at referred appointments will be facilitated by the study, including any attendant transportation costs to access off-site services. Any psychotic symptoms and risks identified during the trial will be discussed with the project mental health professional and women will be referred for specialist mental health care if required. Women assessed as eligible to participate at the time of screening but who do not consent to take part will receive the same stepped mental health care model, depending on their symptoms and any imminent risks.

Otherwise eligible women reporting no past-year IPV exposure will be invited to participate in the separate, nested feasibility trial of standard PST for depression but will not be eligible for this study of PST-IPV.

### Participant consent

Interested women who cannot read will be read aloud the information sheet. If unable to write, participants will record a thumb print, signed by a literate witness (a high school-educated assistant) after confirming that the information sheet has been read aloud correctly; otherwise, participants will provide written informed consent. Study documents will be kept in the locked research office but will be accessible to women at their request. The information sheet includes details of how participants can access compensation, if required.

### Participant reimbursement

All participants will be reimbursed for their time attending research interviews in addition to transport costs and any additional expenses incurred. Participants will be reimbursed for transport costs to attend PST or PST-IPV sessions but will not be compensated for their time during these sessions. This is to determine the acceptability of psychological intervention sessions to women and enable calculation of drop-out rates. A fund will be available to assist participants disclosing IPV who wish to access support services (for example. to facilitate transport to a government social support office or police station).

### Randomisation and allocation to trial arms

Research staff will telephone the Centre for Innovative Drug Development and Therapeutic Trials for Africa data manager, based at the College of Health Sciences, Addis Ababa University, Ethiopia, who is not otherwise involved in this study, to allocate each participant to a study arm (PST-IPV, standard PST or enhanced usual care) using a random number list generated by the trial statistician (GM), who is not otherwise involved in data collection processes. The data manager will telephone a separate member of research staff based in Silt’e, who will inform a clinician trained to deliver PST-IPV or standard PST when a new participant has been allocated to that trial arm. Research staff will provide participants with an unmarked card indicating their enrolment in the trial and agree a date and time to attend their first session of PST-IPV or standard PST. When they attend, they will be expected by their allocated clinician. For participants allocated to enhanced usual care, their antenatal care provider will provide them with information about sources of support and the research assistant will arrange a follow-up appointment 9 weeks later.

Women who do not attend their appointed date and time will be contacted using the telephone number (where applicable) and/or household details they have provided at trial enrolment or through their allocated HEW, up to a maximum of three attempts.

### Masking

Given qualitative differences between PST and PST-IPV, clinicians delivering the intervention cannot be masked to participant allocation. To avoid contamination, PST-IPV, standard PST and enhanced usual care will be delivered by different practitioners who have either attended a PST-IPV or a standard PST training course, or received basic training to provide enhanced usual care. Post-intervention outcome assessments will be conducted by trained, independent assessors (working in separate offices from practitioners delivering interventions and not travelling together) masked to intervention allocation, with a minimum post-high school diploma level of education. Independent assessors will document any incidents of unmasking. Data analysts will also be masked to intervention allocation.

### Intervention arms

Participants randomised to PST-IPV or standard PST will attend four intervention contacts within a maximum of 8 weeks. Where birth occurs before the end of treatment, the feasibility of continuing PST-IPV or standard PST post-natally will also be assessed.

Both manualised interventions will be delivered by trained health workers, such as nurses and midwives, supervised by Psychology Masters-level qualified research staff, with access to a project mental health professional for clinical concerns. Responses to concerns and risk incidents will follow standard operating procedures (SOPs). Government-employed health workers trained to deliver the interventions will be paid for their time.

Both PST-IPV and standard PST intervention arms follow the same structure of four sessions, based on the model successfully employed in South Africa and adapted for this rural Ethiopian setting [27, 66]. Session 1 focuses on basic psychoeducation, introduction to PST, identifying the most important things in the woman’s life and categorising problems into three groups. Session 2 focuses on revising session 1, coping strategies for ‘group A’ problems (which are upsetting but do not influence the most important things in the woman’s life) and the six-step problem solving method for ‘group C’ problems (which are important and can be solved). Session 3 focuses on revising session 2, coping strategies for ‘group B’ problems (which are important but cannot be solved) and psychoeducation about the phases of coping with bereavement and loss. Session 4 focuses on revising session 3, using problem solving skills in everyday life and reviewing how the coping strategies worked in practice. All sessions involve assigning and reviewing take-home activities.

PST-IPV content and materials are adapted to address the needs and experiences of women affected by IPV, whilst standard PST content and materials are generic. Adaptations for women experiencing IPV include training staff using content and materials from the new WHO curriculum on caring for women subjected to violence [67], attention to safety and sensitivity where women list IPV-related problems during sessions (including training with worked examples of problem solving focused on IPV), and adaptation of PST case studies to reflect common problems associated with IPV.

Participants randomised to PST-IPV or standard PST will attend a total of six contacts: one baseline pre-intervention research assessment, four intervention contacts and a follow-up research assessment contact 9 weeks after recruitment. The feasibility of follow-up 9 weeks after recruitment will be assessed and may change, depending on gestation of pregnancy at the time of enrolment.

### Enhanced usual care arm

One-third of participants will be randomised to information only about sources of support (enhanced usual care; see Supplementary file 2). They will attend two contacts: one pre-intervention assessment at which they are provided with information about relevant sources of support, and one follow-up assessment 9 weeks later.

In our meta-analysis we found that randomisation of control group participants with depression, anxiety, PTSD or psychological distress symptoms and IPV exposure to waiting list, treatment as usual, or enhanced usual care arms is widespread [21]. This, coupled with the lack of any standard intervention provision for perinatal depression and/or IPV in rural Ethiopia and the provision of safeguards in the study design, justify the use of a control arm, which comprises enhanced usual care (provision of information about sources of support).

### Measurement: participants

**Fig. 2:**
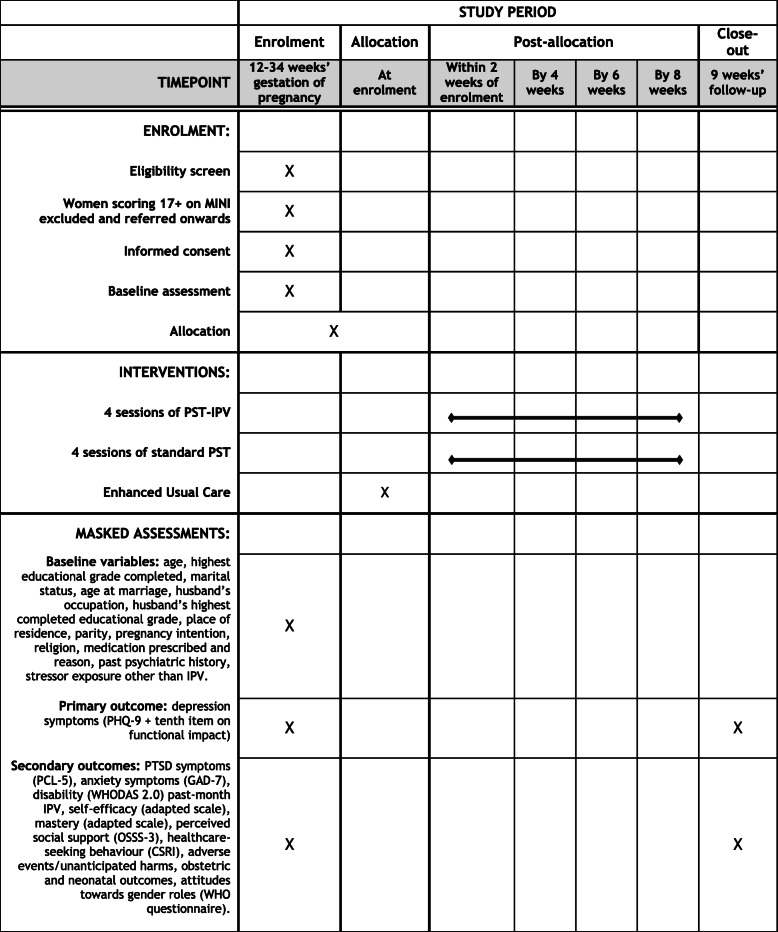
Schedule of participant enrolment, interventions and assessments. CSRI client service receipt inventory, GAD-7 Generalised Anxiety Disorder 7 scale, IPV intimate partner violence, MINI Mini-International Neuropsychiatric Interview, OSSS-3 Oslo Social Support Scale 3, PCL-5 post-traumatic stress disorder checklist for Diagnostic and Statistical Manual version 5, PHQ-9 Nine-item Patient Health Questionnaire, PST problem solving therapy, PTSD post-traumatic stress disorder, WHO World Health Organization, WHODAS World Health Organization Disability Assessment Schedule

Women will be assessed at baseline and then 9 weeks later in a private room of the health care facility at a time convenient to them (Fig. 2). During these assessments, fully structured measures will be administered to consenting participants in an interview format. The time taken to complete planned questionnaires will be tested prior to commencing the study. Where administration of study measures takes longer than 1 h per session or is experienced by research staff or participants as unduly burdensome, instruments will be removed from the assessment questionnaire. This information will contribute to the study’s process evaluation.

#### Primary participant outcome

At baseline and the independent, masked, 9-week follow-up interview, depression symptoms will be measured using the Amharic-translated PHQ-9 [59], a nine-item questionnaire asking about the presence of depressive symptoms in the preceding 2 weeks. Each item is rated according to persistence of the symptom (0 = not at all, 1 = several days, 2 = more than half of the days, 3 = nearly every day). PHQ-9 scores pre- and post-participation will be compared to calculate the mean difference in depression symptom reduction in PST-IPV, standard PST and enhanced usual care arms. Proportions of participants showing a treatment response, defined as a 50% reduction in PHQ-9 score, will also be calculated, although this feasibility trial will not be powered to detect treatment efficacy.

#### Secondary participant outcomes

At baseline and 9-week follow-up, the following will be measured using Amharic-translated scales. If scores are normally distributed, mean differences will be compared between arms using *t* tests and, if not normally distributed, median differences will be compared between arms using non-parametric statistical tests.

- Post-traumatic stress symptoms using the PTSD checklist for the Diagnostic and Statistical Manual version 5 (PCL-5) [68] which has been adapted for a rural Ethiopian context
- Anxiety symptoms using the Generalised Anxiety Disorder 7 (GAD-7) scale [69]
- Disability using the 12-item Ethiopian adaptation [70] of the WHO Disability Assessment Schedule (WHODAS 2.0) [71]
- IPV: participants will be asked about past-month IPV at baseline and 9-week follow-up to determine whether the frequency of different types of IPV (physical, sexual, psychological, coercive control) change during the study period
- Self-efficacy using an adapted self-efficacy scale validated in Ethiopia for sexual health research [72]
- Mastery (the extent to which the person considers themselves in control of forces affecting their lives in important ways [73]) using a 15-item multi-cultural mastery scale [74] adapted for rural, non-Western communities
- Perceived social support using the Oslo Social Support Scale 3 (OSSS-3) [75] previously used in this region in a study of antenatal depressive symptoms [46]
- Health care-seeking behaviour using the client service receipt inventory [76] adapted for Ethiopia [77] and modified to focus on the past month; the client service receipt inventory will be used to quantify the frequency of primary health care visits, inpatient admissions, private sector (including traditional healer) contacts, medications and investigations of women in each arm, in the past month, pre- and post-participation
- Adverse events or unanticipated harms taking place during the study period
- Obstetric and neonatal outcomes: HEWs, who maintain maternal care records, will report the location of birth and any stillbirths and early neonatal deaths among study participants, attendance at subsequent ANC appointments and medication prescriptions for physical and mental health, as they are not reliably self-reported [47]; where women attend ANC at a combination of HEW health posts, health centres and/or primary hospitals, antenatal contacts at all relevant services will be collected; the feasibility of collecting this data will be explored during analysis
- Attitudes towards gender roles using the WHO Attitudes Towards Gender Roles questionnaire [42]

### Measurement: process evaluation

#### Assessing parameters and feasibility for a future trial

The mixed-method process evaluation will assess variables associated with intervention implementation processes. Recommended intervention process outcomes (for implementation) include acceptability, adoption, appropriateness, feasibility, fidelity, implementation cost, penetration and sustainability [78]. In this feasibility trial, we will focus on evaluating:

- Acceptability of PST-IPV and of the study design to women and health workers in terms of uptake, completion and drop-out rates
- Adoption by health workers
- Appropriateness from women and health workers’ perspectives
- Feasibility in terms of practical organisation
- Fidelity of delivery
- Cost
- Safety

The following administrative data will be collected, which will inform the feasibility outcome measures shown in brackets:

- Screening rates and reasons for refusal (acceptability)
- Recruitment rates (acceptability, feasibility)
- Numbers of eligible women at each clinical site (appropriateness)
- Proportions of eligible women agreeing to participate (acceptability)
- Willingness to be randomised and comprehension by participants of randomisation (acceptability of allocation)
- Session duration (appropriateness, feasibility, fidelity)
- Take-home activity completion (acceptability, fidelity, appropriateness)
- Drop-out rates (acceptability, feasibility)
- Follow-up rates (feasibility of retention)

Research staff will document key site features to enable comparison of process data, and keep field journals noting:

- Staff willingness to participate at each site and retention in the study (acceptability, adoption, appropriateness, feasibility)
- Optimal recruitment procedures (feasibility)
- Acceptance of randomisation at each site (acceptability, appropriateness, feasibility)

#### Qualitative interviews

The acceptability and burden on women and staff of the intervention itself (including which components are experienced as helpful or unhelpful), randomisation, outcome measures and follow-up will be assessed through qualitative interviews with a purposively sampled sub-group of approximately six women (three per intervention arm) and six staff (three per intervention arm). Interviews will also explore feasibility and acceptability of the study’s SOP, which dictates the management of safety, confidentiality and ethical concerns, risk disclosures during sessions and research contacts. Interviews will be conducted in Amharic by research staff who did not have extended contact with participants during the trial in a private location convenient to them. Participants will be compensated for their time, and interviews will be audio-recorded, transcribed and translated into English.

#### Intervention fidelity and supervision

Health workers trained to deliver PST-IPV and standard PST will receive monthly supervision with a local psychiatric nurse. Health workers will audio-record PST-IPV and standard PST sessions with the participant’s consent for review by their supervisor and to inform discussions of challenging cases during supervision sessions.

A random sample of these audio recordings will be evaluated using the ENACT scale [79] to assess therapist competence (quality) alongside supervisor observations and therapist logs. ENACT has been adapted for the Ethiopian context and has been shown to be reliably administered by trained clinicians [80].

Recordings will also be evaluated for intervention fidelity and session completion using a checklist of session components. Supervision records and therapist logs will also be reviewed for intervention fidelity and contamination between arms [27]. The frequency and duration of IPV-specific content during recorded sessions of both PST-IPV and standard PST documented in therapist logs will also be noted to determine the extent to which participants raise and explore IPV-related problems during sessions. The quality of therapist–client rapport will be assessed using the Amharic-translated helping alliance questionnaire [81], which has been tested in this setting [82].

Outcome assessors will receive supervision from a Psychology Masters-qualified supervisor. Supervision logs will include discussing any items which are poorly understood or completed, and reviewing the time taken for outcome assessment to assess its acceptability and feasibility.

### Statistical analysis

We will follow the Consolidated Standards of Reporting Trials (CONSORT) extension to randomised pilot and feasibility trials [83].

We will review process evaluation data for indications of sub-group differences in uptake, recruitment and retention of participants by site, health worker, recruitment method/site, participant (age, education level, religion, parity) and health worker characteristics (age, profession, years of experience). Quantitative scale results will be analysed using STATA [84].

Feasibility studies are not powered to calculate effect sizes but standard deviations and drop-out rates will be calculated on the intention-to-treat sample (all women who were randomised, regardless of uptake of the intervention) to inform sample size calculation for a future RCT, for outcome measures, recruitment rates and drop-out rates. Quantitative process data will determine future RCT design improvements, such as session number and duration, recruitment, outcome measures, and strategies preventing contamination. The extent of missing data will be evaluated as part of the process evaluation. For clinical outcome measures, missing data will not be imputed. Rather, standard deviations will be calculated using data from participants for whom outcome measures are available.

We will apply the framework approach to thematic analysis of qualitative interviews [85] and triangulate the results with quantitative process data through researcher meetings. The full descriptive analysis will include contextual barriers and facilitators influencing study outcomes.

### Ethical considerations

Strategies to ensure data protection, quality assurance and dissemination of results are outlined in Supplementary file 3 and data collection forms are provided in Supplementary file 4. Ethical approval has been provided by the Institutional Review Board of the College of Health Sciences, Addis Ababa University, Ethiopia (protocol number: 032/19/CDT) and the Psychiatry, Nursing & Midwifery subcommittee of King’s College London’s College Research Ethics Committee, UK (reference: HR-18/19–9230); see Supplementary file 5.

This study involves recruitment of potentially vulnerable women, disclosure of IPV, and detecting depression, anxiety and PTSD symptoms. The SOP therefore outlines actions to mitigate potential risks. If participants disclose current exposure to IPV, research staff will be trained to listen non-judgementally, offer privacy and confidentiality, and information about local agencies who can provide assistance (see Supplementary file 2) [42] in keeping with WHO guidelines, irrespective of the study arm.

#### Sensitivity

Female research staff will be carefully selected with the sensitivity of subject matter in mind. Once recruited, they will receive locally tailored IPV training [44]. When approaching women, great sensitivity will be exercised. In all cases, care will be taken to ensure that women do not feel pressurised to participate. We will ensure anonymity of interview transcripts and any quotations used in publications or reports.

#### Safety

The SOP outlines measures to mitigate potential risks to participants, including abusive partners learning of their involvement. These include a protocol for responding to concerns or disclosures of risk, emergency contact information, conduct when communicating with vulnerable participants, documenting and responding to serious adverse events and reporting them to the King’s College London and Addis Ababa University institutional review boards which have provided ethical approval for this trial. Numbers of serious adverse events will be calculated per trial arm. A data monitoring committee will not be instituted due to the small sample size of this feasibility trial, which is not powered for interim analyses.

#### Risk of harm

The risk of harm to participants will be minimised by providing full information about the study prior to voluntary participation. Participants are free to withdraw from taking part at any time without needing to give a reason. Their individual data can be withdrawn from the randomised feasibility trial until the final data collection, at which point analysis will commence.

Some participants might become distressed when speaking about their mental health or experiences of IPV. Questions will be asked and worded sensitively to minimise this occurring during research assessments. During sessions of PST-IPV, the opportunity to express emotion about problems and difficulties may be therapeutic, however. Clinicians will be trained in responding to distress. Data collectors will be trained to be sensitive to signs of distress and when to suggest rescheduling or discontinuing an interview. If the participant remains distressed, the data collector will contact their supervisor and arrange appropriate support. If needed, women can be referred to their ANC provider, who has been trained in primary mental health care and the situation can be discussed with the project mental health professional.

## Discussion

Despite being an important social determinant of physical and mental health, there is limited evidence for brief, task-shared psychological interventions adapted to address the mental health needs of women experiencing IPV in LMICs. Interventions tailored to the specific LMIC context and adapted to address the experiences of women affected by IPV require development, evaluation and implementation. This randomised feasibility trial comparing PST-IPV with standard PST and enhanced usual care in rural Ethiopia and mixed-methods process evaluation will determine feasibility and acceptability to women and health workers to inform the design of a future RCT. It will generate hypotheses, explore causal mechanisms and contextual factors relevant to mental health clinicians, researchers and implementation scientists in primary care and ANC in LMICs, which can be investigated across contexts in a future, fully powered multi-centre RCT.

### Strengths and challenges

This is the first study of its kind in this rural Ethiopian setting. Most research into psychological interventions for anxiety and depression in LMICs comes from urban or peri-urban areas of middle-income countries, and only two to date have been adapted for women experiencing IPV [22, 23]. Neither of these interventions trialled a brief intervention, embedded within ANC in a rural, low-income country setting. The results will be informative to researchers developing brief psychological interventions adapted for this and other resource-restricted settings.

This three-arm randomised feasibility trial benefits from a shared control group with a separate, nested study of standard PST. The efficiency of shared control groups is well documented [86] and multi-arm trials are recognised for their simplicity, speed and reduced cost relative to two-arm trials [87]. The study focus on evaluating and refining the feasibility of both the PST-IPV intervention and the study design using a mixed-method process evaluation ensures that improvements will be made prior to a definitive RCT.

The sensitivity of discussing mental health and IPV, logistical challenges to women’s participation in research, limited education and literacy and competing priorities on ANC staff time are all anticipated challenges to the successful completion of this study. These will be mitigated by stringent ethical conduct emphasising confidentiality and supporting women at risk of IPV, supporting women to access intervention arms, adapting recruitment and intervention procedures to accommodate variable education and comprehensive training and supervision of health workers. However, unforeseen geopolitical eventualities may arise in rural, low-income settings which compromise the conduct and completion of this protocol as planned. The benefit of this feasibility trial is to identify study design problems as early as possible in order to mitigate their impact on the resultant research evidence.

## Trial status

This is protocol version 1.0. This trial was prospectively registered on the Pan-African clinical trials registry (PACTR202002513482084) on 13 December 2019. Recruitment of participants has not yet commenced. Recruitment of participants is anticipated to complete by 31 December 2020. Any proposed changes to the protocol will be submitted to King’s College London and Addis Ababa University ethics review boards and updated on the pan-African clinical trials registry. Research staff will inform trial participants, where required, and changes will be discussed in the ultimate results publication. The trial sponsor is King’s College London, De Crespigny Park, Denmark Hill, London SE5 8AF, UK.

## Supplementary information

**Additional file 1: Supplementary file 1.** Provisional theory of change map for PST-IPV. **Supplementary file 2.** Example information sheet about local sources of support provided to all participants. **Supplementary file 3.** Data protection, quality assurance and dissemination plans. **Supplementary file 4.** Data collection forms. **Supplementary file 5.** Ethical approval. **Supplementary file 6. (a)** Participant information sheet. **(b)** Participant consent form. **(c)** Health worker information sheet and health worker consent form.

## Data Availability

The minimum dataset used to calculate the study findings will be included with the study’s resultant outcome reporting manuscript as a supplementary file. Additional datasets generated during this study will be available from the corresponding author upon reasonable request.

## Abbreviations

ANC: Antenatal care
CBT: Cognitive behavioural therapy
HEW: Health extension worker
IPV: Intimate partner violence
LMICs: Low- and middle-income countries
PHQ-9: Nine-item Patient Health Questionnaire
PRIME: Programme for improving mental health care study
PST: Problem solving therapy
PTSD: Post-traumatic stress disorder
RCT: Randomised controlled trial
SOP: Standard operating procedure
WHO: World Health Organization
