# Supplementary material for: Problem solving therapy (PST) tailored for intimate partner violence (IPV) versus standard PST and enhanced usual care for pregnant women experiencing IPV in rural Ethiopia: protocol for a randomised controlled feasibility trial
Source: Trials. 2020 Jun 1;21:454. doi: 10.1186/s13063-020-04331-0 (PMC7268746; doi:10.1186/s13063-020-04331-0)
Supplement: Supplementary file 1 — Additional file 1: Supplementary file 1. Provisional theory of change map for PST-IPV. Supplementary file 2. Example information sheet about local sources of support provided to all participants. Supplementary file 3. Data protection, quality assurance and dissemination plans. Supplementary file 4. Data collection forms. Supplementary file 5. Ethical approval. Supplementary file 6. (a) Participant information sheet. (b) Participant consent form. (c) Health worker information sheet and health worker consent form. [file 13063_2020_4331_MOESM1_ESM.docx]

**SUPPLEMENTARY FILE 1: Provisional Theory of Change map for PST-IPV**

**Abbreviations**

ANC: Antenatal care, HF: Health facility, HW: Health worker, e.g. midwife, ANC nurse, IPV: Intimate partner violence, mhGAP: WHO mental health gap action programme, MINI: mini international neuropsychiatric inventory, PST-IPV: Problem solving therapy adapted for women experiencing IPV, RA: Research assistant, RCT: randomised controlled trial, SOP: standard operating procedure

Reduced IPV

Improved treatment of perinatal depression

Improved identification of perinatal depression and IPV

Women meeting inclusion criteria invited to attend

**Assumptions**

Women able to attend sessions, women motivated to attend all sessions, delivery in HCs acceptable to HWs and women, PST-IPV model and training acceptable to HWs and women, trained staff available, supervisors available, no problems with childcare, room available for screening and sessions

**Additional Interventions**

^1^ Share results with stakeholders following study completion ^2^mhGAP training of all staff, ^3^Training of supervisor(s), supervision provision, ^4^Reinforcement of attendance and alignment with ANC, ^5^Comprehensive adaptation process

**Barriers**

Mobility of women, stigma among women and HWs, difficulty of women to attend despite transport cost provision, external factors, e.g. life events, unrest, staff work burden

**Impact**

**Long-term outcomes**

**Intervention delivery and intermediate outcomes**

**Engagement and Set-up**

Information session in each HF^2^

Eligible women identified and screened in ANC

Practicalities discussed with HF managers beforehand

Collaborative working between PST-IPV coordinators and HF HWs

Attendance at training and retention in PST-IPV delivery by selected staff

PST-IPV aligned with HW and women’s priorities in qualitative interviews and Theory of Change workshops

Community advisory board informed and updated^1^

Women’s depressive symptoms improve (**primary outcome**)

Increased confidence of health workers to ask about and address depression and IPV

Increased knowledge about IPV and self-help for perinatal depression among women and HWs

Improved self-efficacy, mastery and perceived social support in women (**hypothesised mediators**)

Increased community awareness of depression and IPV^1^

HWs deliver PST-IPV as per training and manual **(fidelity**)

Supervisors monitor and support staff to deliver PST-IPV (**quality, fidelity**)^3^

Women receive a course of PST-IPV

Staff escalate suicidality (MINI) and discuss risk concerns as per SOP

(**safety, ethics**)

Better engagement with and attendance at ANC^4^

Development of a scalable, feasible intervention for depression and IPV in this setting

More compassionate, respectful maternal care

**SUPPLEMENTARY FILE 2: Example information sheet about local sources of support provided to all participants**

| **Office (ቢሮ)** | **Contact person**  **(የሚያገኙት ግለሰብ)** | **Position**  **(የስራ መደብ /ሀላፊነት)** | **Contact number**  **(ስልክ)** |
| --- | --- | --- | --- |
| Police (ፖሊስ) |  |  | 0468830xxx |
| Women and Child Affairs (የሴቶችና ህፃናት ) | X  X | Head (ሀላፊ)  Coordinator (አስተባባሪ) | 0468830xxx  091xxxxxxx  091xxxxxxx |
| Youth (ወጣቶች) | X | Coordinator (አስተባባሪ) | 0468830xxx  091xxxxxxx |
| Health Bureau (ጤና ቢሮ) | X | Head (ሀላፊ) | 0468830xxx  091xxxxxxx |
| District court/Justice  (ወረዳ ፍትህ/ፍርድቤት) | X | Women and Child Officer (ፍርድ ቤት) | 0468830xxx |
|  | X | Justice Head (ወረዳ ፍትህ) | 0468830xxx |

**SUPPLEMENTARY FILE 3: Data Protection, Quality Assurance and Dissemination Plans**

New data collected during this study will comprise:

- - Paper documents (e.g. consent forms, notes taken during interviews, PST-IPV and standard PST session documentation).
  - Audio recordings and electronic transcriptions (of Amharic language interviews, with English translation).

All participant names and details and paper documents will be stored in a locked filing cabinet or cupboard in a secure office in Addis Ababa University (AAU). Data documented on paper will be double-entered using EpiData software by trained research staff. All participants will be allocated a pseudonymous participant project identifier, which will be used instead of a name on all subsequent documentation. Qualitative transcripts mentioning names and other identifying details will be pseudonymised. Where a single electronic file is required to match participant names to their pseudonymised identifiers, this will be password-protected, saved on a password-protected cloud or hard drive, and not shared outside staff members who require the information for research conduct purposes. Electronic documents will be saved on a password-protected computer hard drive. An encrypted cloud and hard drive will be used to provide back-up data storage. If electronic documents need to be transferred, for example between offices at AAU and KCL, an encrypted method and password protection will be used. Only members of the research group, all of whom will have received Good Clinical Practice training, will have access to the data. Audio files will be encrypted, password-protected and only identifiable by the participant’s project identifier. Participant names will not be stated in recordings.

Personal data will be destroyed two years after completion of data analysis. Other research data will be destroyed seven years after completion of data analysis. Following completion of this PhD research, electronic records will be archived on a secure, password-protected drive, to facilitate potential future use.

Data collectors will be female staff with a minimum education of grade 10, who have been trained in administration of all measures using interactive training techniques, including role plays and observation of practice interviews. Research staff will be trained about discussing IPV, informed by WHO and Program for Appropriate Technology in Health guidance on study design, field worker training, ethics and safety in IPV research.(93)

A research supervisor will oversee data collection, review questionnaires for incompleteness or inconsistency at the time of data collection and ensure ethical procedures are followed. The study site will be visited regularly by senior investigators, to ensure that ethical principles are adhered to, including random checks of procedures for obtaining informed consent. Monitoring will be supported by established procedures for ethical data management.

The community advisory board of the ASSET (health system strengthening in sub-Saharan Africa) study in which this PhD research is nested includes representatives from the district health office and other relevant district offices, in addition to service users, health centre heads, religious leaders, non-governmental organisation and community leaders. The results of this trial will be shared with the board once analysis concludes, as well as to community stakeholders identified by a previous mapping exercise. A policy brief will be prepared, to inform Ministry of Health officials, with whom ASSET works in active partnership. Trial results will be published in a peer-reviewed journal and presented at local and international conferences. All authors of resultant publications will meet International Committee of Medical Journal Editors criteria for authorship. No professional writing services will be used.

**SUPPLEMENTARY FILE 4: Data Collection Forms**

## Baseline data collection

| 001 | Pseudonymised identifier |  | |
| --- | --- | --- | --- |
| 1 | Timing of first attendance for antenatal care | ____ ____ weeks gestation | |
| 2 | Number of current ANC visit | 1^st^ visit | 1 |
|  |  | 2^nd^ visit | 2 |
|  |  | 3^rd^ visit | 3 |
|  |  | 4^th^ visit | 4 |
|  |  | 5^th^ or more visit | 5 |
|  |  | No documentation | 7 |
| 3 | Gravida | ____ ____ | |
| 4 | Parity | ____ ____ | |
| 5 | Gestation | ____ ____ weeks | |
| 6 | Any current problems | 1.  2.  3. | |
| 7 | Documentation of past history of mental health problems | Yes, documented history of mental disorder | 1 |
|  |  | Yes, documented that no history of mental disorder | 2 |
|  |  | No documentation | 7 |
| 8 | Documentation of current mental health problems | Yes | 1 |
|  |  | No | 0 |
|  |  | No documentation | 7 |
| 9 | Documentation of current violence exposure | Yes | 1 |
|  |  | No | 0 |
|  |  | No documentation | 7 |
| 10 | ANC appointment attendance | All attended | 1 |
|  |  | Some attended | 2 |
|  |  | None attended | 3 |
|  |  | No documentation | 5 |
| 11 | Medications prescribed | Physical health | 1 |
|  |  | Mental health | 2 |
|  |  | Both | 3 |
|  |  | None prescribed | 4 |
|  |  | No documentation | 7 |
| 12 | Obstetric outcome/complications | Haemorrhage | 1 |
|  |  | Obstructed/prolonged labour | 2 |
|  |  | Sepsis | 3 |
|  |  | Miscarriage | 4 |
|  |  | (Pre-)eclampsia | 5 |
|  |  | Ruptured uterus | 6 |
|  |  | No documentation | 7 |
| 13 | Neonatal outcome/complications | Live birth without complications of prematurity | 1 |
|  |  | Live birth with complications of prematurity | 2 |
|  |  | Stillbirth (late foetal death at/after 24 weeks at/before delivery) | 3 |
|  |  | Missed abortion (early foetal death before 24 weeks gestation with retention of foetus) | 4 |
|  |  | Early neonatal death | 5 |
|  |  | Spontaneous abortion | 6 |
|  |  | No documentation | 7 |

**Sociodemographic characteristics**

| 1 | How old are you? | ____ ____ years | |  |
| --- | --- | --- | --- | --- |
| 2 | Have you received any education? | No formal education | 1 |  |
|  |  | Primary education only | 2 |  |
|  |  | Secondary education only | 3 |  |
|  |  | Post-secondary education | 4 |  |
| 3 | What is the highest educational grade that you have reached? | ____ ____ grade |  |  |
| 4 | Which sub-district (kebele) do you live in? |  | |  |
| 5 | What is your marital status? | Single (never married) | 1 |  |
|  |  | Separated | 2 |  |
|  |  | Divorced | 3 |  |
|  |  | Widowed | 4 |  |
|  |  | Monogamous marriage | 5 |  |
|  |  | Polygamous marriage | 6 |  |
| 6 | (If married) at what age did you marry? | ____ ____ years |  |  |
| 7 | What is your religious affiliation? | Orthodox Christian | 1 |  |
|  |  | Muslim | 2 |  |
|  |  | Protestant | 3 |  |
|  |  | Catholic | 4 |  |
|  |  | None | 5 |  |
|  |  | Other (specify) | 6 |  |
| 8 | What is your husband’s occupation? |  |  |  |
| 9 | Has your husband received any education? | No formal education | 1 |  |
|  |  | Primary education only | 2 |  |
|  |  | Secondary education only | 3 |  |
|  |  | Post-secondary education | 4 |  |
| 10 | Was your current pregnancy planned? | Yes | 1 |  |
|  |  | No | 0 |  |
| 11 | If Yes, did this pregnancy occur in your scheduled period? | Yes | 1 |  |
|  |  | Would have preferred it to happen later | 0 |  |
| 12 | List of Threatening Experiences questionnaire Total Score: | ____ ____ |  |  |

## Screening: Patient Health Questionnaire (PHQ-9)

###

### MINI Suicidality Scale

## Screening: Intimate Partner Violence Measures

###

### Non-Graphic Language Screening Questions

###

### WHO Multi-country Survey: IPV Questions

##

## PCL-5

## GAD-7

## World Health Organization Disability Assessment Schedule (WHODAS-12)

##

## Adapted self-efficacy scale

## Translated Multicultural Mastery Scale

## Oslo Social Support Scale (OSSS-3)

## Attitudes Towards Gender Roles (WHO Multi-Country Study)

##

## Modified Client service receipt inventory (CSRI)

## Enhancing Assessment of Common Therapeutic factors (ENACT) rating scale

## Helping Alliance Questionnaire

**SUPPLEMENTARY FILE 5: Ethical Approval**


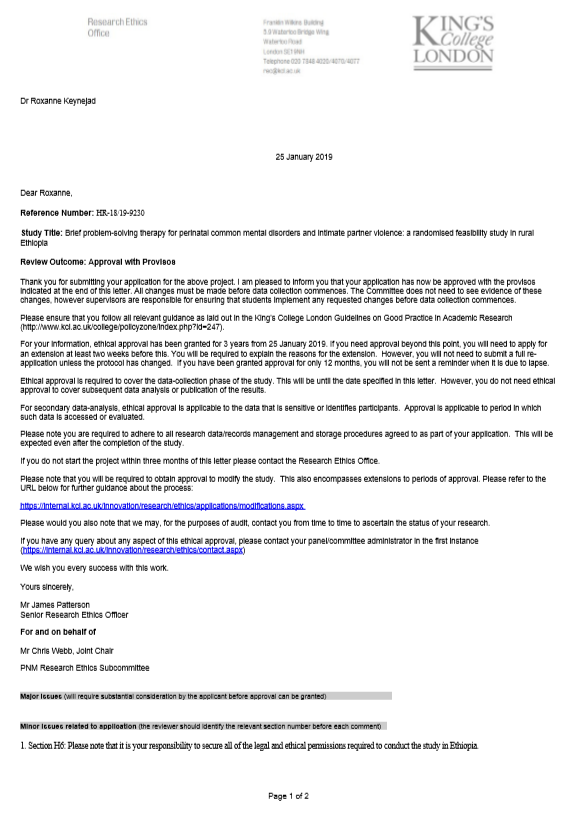


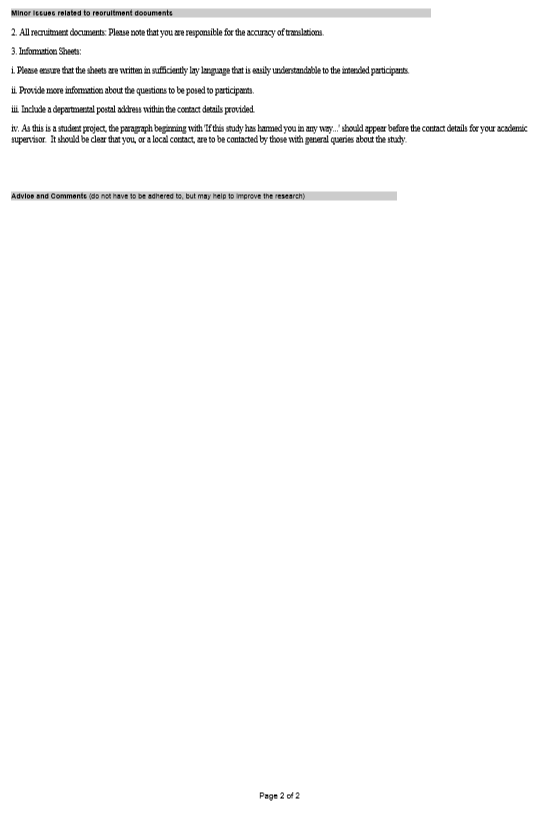


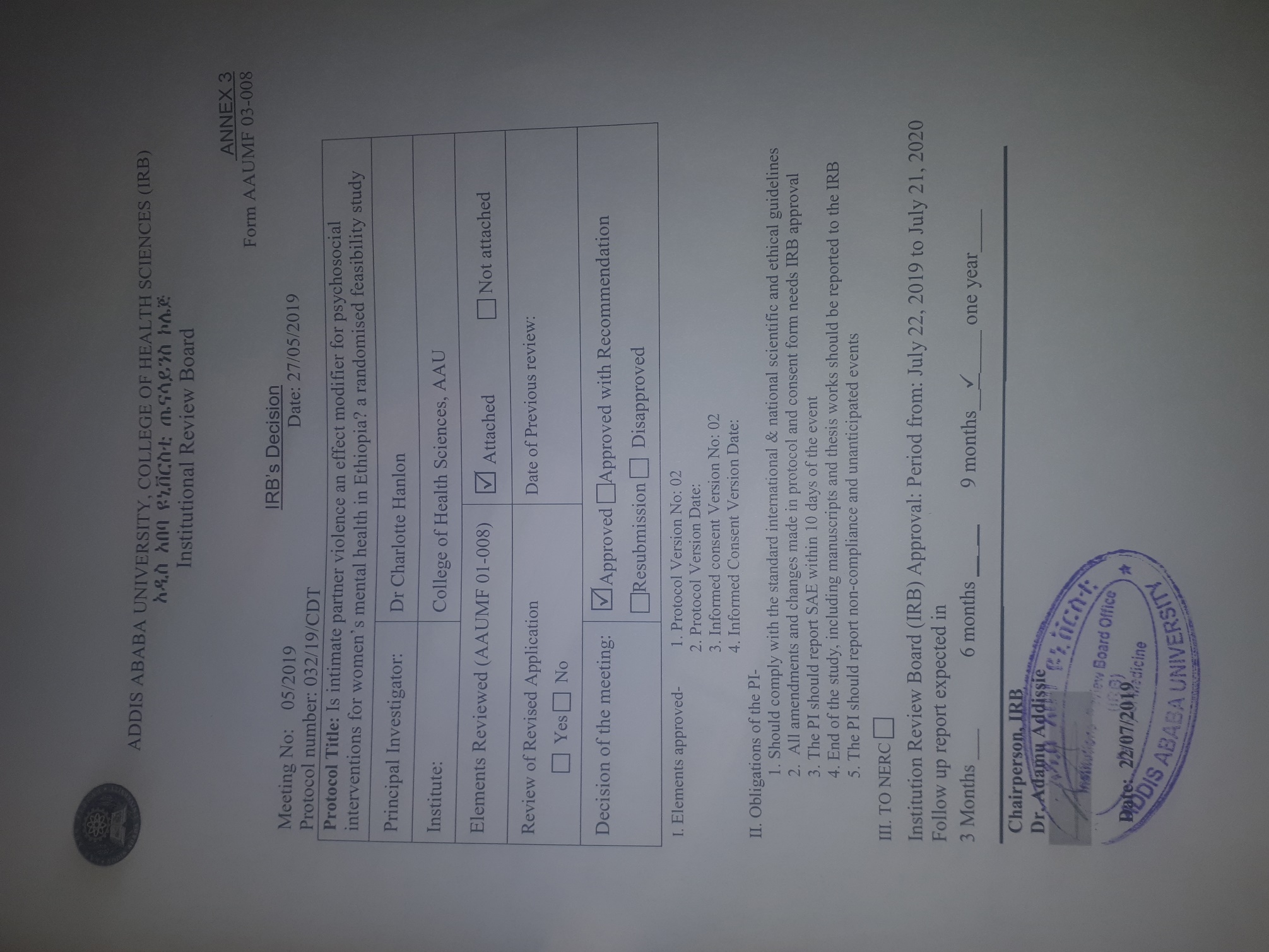


**SUPPLEMENTARY FILE 6a: Participant Information Sheet**

**INFORMATION SHEET FOR PARTICIPANTS**

*
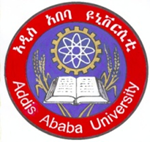
*
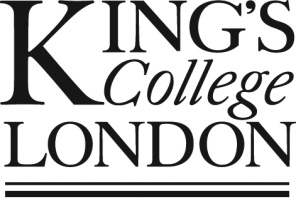


*KCL REC Ethical Clearance Reference Number:* HR-18/19-9230

*AAU IRB Ethical Clearance Reference Number: 032/19/CDT*

**YOU WILL BE GIVEN A COPY OF THIS INFORMATION SHEET.**

**TO PRESERVE CONFIDENTIALITY WE ASK THAT IT BE KEPT IN OUR LOCKED RESEARCH OFFICE.**

**Title of study:** A feasibility study of problem-solving therapy for depression and intimate partner violence in Ethiopia.

We would like to invite you to take part in our research project. This study is a collaboration between researchers from Addis Ababa University (AAU) and King’s College London (KCL) in the United Kingdom (UK) and University of Cape Town (UCT) in South Africa. You should only participate if you want to; choosing not to take part will not disadvantage you in any way. Before you decide whether you want to take part, it is important for you to understand why the research is being done and what your participation will involve. Please take time to read the following information carefully or have the information read out to you and discuss it with others if you wish. Ask us if there is anything that is not clear or if you would like more information.

**What is the purpose of the study?**

Stress, distress, feeling sad and becoming depressed are common experiences, including in pregnancy. Many women also experience violence in their relationship with their husband or partner, which is called ‘intimate partner violence’ (IPV). We have adapted a short talking treatment for depression called problem-solving therapy (PST) for Ethiopia. The version of problem-solving therapy which has been adapted to address IPV is called PST-IPV. This study will compare three different options for pregnant women with depressive symptoms. The purpose of this study is to find out if problem-solving therapy, problem-solving therapy adapted to address IPV and information can be compared in a randomised research study and to explore how acceptable and possible it is to provide them in this part of Ethiopia.

**Why am I being invited to take part?**

You are being invited to take part in this study because you are between 12 and 34 weeks pregnant, intend to remain living in this area for the next three months, and speak Amharic. You will be asked questions to understand whether you are experiencing stress or distress that is affecting your life. You will be invited to take part in a research study if so.

**Do I have to take part?**

Participation is voluntary: you do not have to take part in this study. You should read this information sheet and if you have any questions you should ask the research team. If you decide to take part, you will be free to withdraw from the study at any time without needing to give a reason. Taking part or not taking part in this study will not affect your healthcare in any way. If you decide to take part we will ask you to sign (or thumb-print) a (witnessed) consent form and you will be given a copy of this consent form; this will be kept in our locked research office to preserve your confidentiality. If you are uncomfortable answering certain questions you can refuse or ask to stop a session or questionnaire at any time, without needing to give a reason.

**What will happen if I take part?**

If you agree to take part, you will speak to the research assistant in a private room in a place and time that suits you best. They will record information about you, including your age, level of education, marital status, religion and so on. They will ask you questions about problems that cause stress in your life, symptoms and how you feel about yourself. We expect this to take about one hour.

You will be **randomised** to one of three possible paths. This means that it is like a lottery that decides which path of the study you will enter: the researchers will not have any control over this and it is not possible for you to choose a particular path.

1. Only if you have experienced intimate partner violence (IPV) in the past year, you may be picked by lottery to have path 1. If so, you will receive problem-solving therapy adapted to address IPV. This means you will receive **four sessions** of problem-solving therapy adapted to consider IPV. This is a brief talking therapy which will be delivered by a trained healthcare professional, who receives supervision from a senior clinical researcher. The four sessions will take place in a private room in a place convenient to you on a day and time that suits you best and last about 30 minutes.
2. If you are picked by lottery to have path 2, you will receive standard problem-solving therapy. This means you will receive **four sessions** of standard problem-solving therapy. This is a brief talking therapy which will be delivered by a trained healthcare professional, who receives supervision from a senior clinical researcher. The four sessions will take place in a private room in a place convenient to you on a day and time that suits you best and last about 30 minutes.
3. If you are picked by lottery to have path 3, you will receive information about sources of support for women experiencing intimate partner violence. You will not receive any problem-solving therapy.

Regardless of which path you are randomised to, if you decide to take part, you will be seen once more by an independent researcher who has not met you before, **nine weeks** after agreeing to take part, and asked to answer some more questions about how you are feeling. We expect this to take about one hour. Your health extension worker will report routine information about your health, pregnancy, appointments and other treatments to us.

A small number of therapy sessions will be audio-recorded to assess the healthcare worker’s technique. You will be asked to give permission for this to take place and are free to say no if you do not wish for your session to be audio-recorded. If you agree, the content of the session will be written down but any information that could identify you will be changed. Once the sessions have been documented, the audio record will be destroyed.

A small number of women who enrol in the study will be asked if they would like to have an audio-recorded interview after the study is completed about their experience. We will explain more about this later if you are chosen. You will be free to say no if you do not wish to have that extra interview.

**What are the possible risks of taking part and how will they be addressed?**

A possible risk of you taking part in this study could be that other people know you are in the study. To avoid this happening, we will therefore take great care to keep your taking part confidential, for example by keeping your copy of this information sheet in our locked research office and organising sessions in a private room in a place and time convenient to you. All of your study information will be recorded with an identification number instead of your name. Paper records will be stored in a secure filing cabinet in our locked research office which has restricted access and electronic records will be password-protected in secure computers or private computer websites.

For some people, when they speak about feeling stress or distress or their husband or partner being violent, this could make them feel upset. If you feel upset during or after any research contacts or sessions of problem-solving therapy or problem-solving therapy adapted to address intimate partner violence, you can stop the interview at any time and the clinical researcher will discuss this with you and offer additional support if you need and want it.

We also take very seriously any risks that we become aware of during the course of the study. If whilst taking part, you identify risks to your or another person’s health or safety, the research assistant will speak to you about how to respond and provide support to you or the person. If there is an imminent danger of serious harm to someone, the police may need to be informed, after discussing this with you.

**What are the possible benefits of taking part?**

If you are picked by lottery to have path 1 or 2 you will receive four sessions of brief talking therapy, which you may find helpful for your health and wellbeing.

If you are picked by lottery to have path 3, you will not receive any sessions but you will receive information about sources of support and will be seen twice by researchers at the start and end of the study.

**Compensation**

You will be compensated for your time attending research interviews (but not sessions of problem-solving therapy or problem-solving therapy adapted to address intimate partner violence) at 50 Birr per contact attended and in addition you will be reimbursed for any travel or other expenses which you incur by taking part in the study.

**What happens if I decide not to take part in this study?**

Your participation in this study is entirely voluntary. Choosing to take part or not to take part in this study will not disadvantage you in any way.

Even after the study has started, you are free to stop taking part at any time, without giving a reason.

If you wish to withdraw from the study, please contact the researchers as described below. You can withdraw your data from the study up until 31/03/2020, after which withdrawal of your data will no longer be possible due to analysis commencing. If you choose to withdraw from the study we will not retain the information you have given thus far, except for demographic information, which we will retain in order to report basic features of participants withdrawing from and remaining in the study.

**Will my taking part be kept confidential?**

Your interview responses will not be identifiable. The fact that you have taken part will be kept confidential. Your responses to questions will be analysed with other participants’ and will not be traceable to you in any reports.

**Data handling**

Your data will be processed in accordance with Ethiopian data protection rules and the United Kingdom’s (UK) General Data Protection Regulation 2016 (GDPR), which exist to protect you and your data. This means that:

Any of your personal data that has been collected will be destroyed two years after data analysis is completed. Following completion of this research, electronic records which are not identifiable to you will be stored on a secure computer which is protected by a password. Anonymised data will be available to future researchers so that we can learn as much as possible, but this will not be identifiable as your information.

Amharic language records will be shared (with your consent) with a transcription and translation service. Any potentially identifiable references made in translated records will be changed to preserve your confidentiality.

As this study is conducted between Ethiopia and the UK, some information that you tell us will be securely transferred between these countries (with your consent). However, your information will not be shared with any parties outside KCL, UCT and AAU. If any information needs to be shared for research, a special transfer agreement will be in place to ensure that information continues to be kept according to Ethiopian data protection rules and UK data protection standards (GDPR).

**Data protection statement**

There will be people responsible for looking after the information you give us, to make sure that the information is looked after properly. We will only ask for information that can help scientific understanding and benefit people in the future ‘in the public interest’. You can give your consent for your personal data to be used in this study by completing the consent form we have provided.

You have the right to access information held about you. To discuss any of these rights or to exercise them, please inform the research assistant or your health worker, who will inform the research assistant. Your right of access can be exercised in accordance with Ethiopian data protection rules and the General Data Protection Regulation.

**Who has reviewed this study?**

The ethics protocol of this study has been reviewed and approved by the Institutional Review Board of the College of Health Sciences of Addis Ababa University (AAU) and by the Psychiatry, Nursing and Midwifery (PNM) Research Ethics Subcommittee (RESC) at King's College London.

**How is the project being funded?**

This research is funded by King’s College London in the UK.

**What will happen to the results of the study?**

Based on your and other women’s responses to this study, we will improve problem-solving therapy adapted to address intimate partner violence (PST-IPV) and the design of this study, to plan a larger study of PST-IPV and standard problem-solving therapy in Ethiopia. The information collected will enable us to design treatments that can be practically provided in this setting in a way that women and healthcare workers find helpful and acceptable.

We will present our findings to other researchers and they will be written about in a PhD thesis and peer-reviewed journal publications. You can request to receive a copy of the English language research from the study researchers (see below). All results will be presented without giving any personal details about you, so other researchers will not be able to identify you.

**What if I have further questions, or if something goes wrong?**

If you would like to talk to someone else about this project, if this study harmed you in any way or if you wish to make a complaint about the conduct of the study, you can contact the Institutional Review Board of the College of Health Sciences of Addis Ababa University, on 0115-5538734 or King's College London: The Chair, Psychiatry, Nursing and Midwifery (PNM) Research Ethics Subcommittee (RESC): [rec@kcl.ac.uk](mailto:rec@kcl.ac.uk)

**If I have some more questions about the research, who can I speak to?**

Please contact Research Assistant Adiyam Mulushoa, telephone number 0910 024757, at the College of Health Sciences, Addis Ababa University, Ethiopia. You can also contact Dr Tesera Bitew on 0911 173 656.

**Thank you for reading this information sheet and for considering taking part in this research.**

**Supplementary File 6b: Participant Consent Form**

**CONSENT FORM FOR PARTICIPANTS IN RESEARCH STUDIES**

Please complete this form after you have read the Information Sheet and/or listened to an explanation about the research.

*
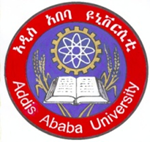
*
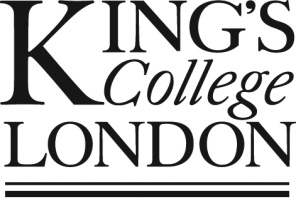


**Title of Study:** A feasibility study of problem-solving therapy for depression and intimate partner violence in Ethiopia

**KCL REC Ethical Clearance Reference Number:** HR-18/19-9230

**AAU IRB Ethical Clearance Reference Number:** 032/19/CDT

Thank you for considering taking part in this research. The person organising the research must explain the project to you before you agree to take part. If you have any questions arising from the Information Sheet or explanation already given to you, please ask the researcher before you decide whether to join in. You will be given a copy of this Consent Form to refer to at any time. To preserve your confidentiality, we ask that it be kept in our locked research office.

**Please tick or initial**

**I confirm that I understand that by ticking/initialling each box I am consenting to this element of the study. I understand that it will be assumed that unticked/initialled boxes mean that I DO NOT consent to that part of the study. I understand that by not giving consent for any one element I may be deemed ineligible for the study.**

**Please tick or initial**

1. I confirm that I have read and understood the information sheet dated 08.01.19, Version 1, for the above study. I have had the opportunity to consider the information and asked questions which have been answered to my satisfaction.
2. I consent voluntarily to be a participant in this study and understand that I can refuse to answer questions and I can withdraw from the study at any time, without having to give a reason, up until 31/03/2020.
3. I consent to the processing of my personal information for the purposes explained to me in the information sheet. I understand that such information will be handled in accordance with the terms of the General Data Protection Regulation and Ethiopian data protection rules.
4. I understand that my information may be subject to review by responsible individuals from Addis Ababa University and King’s College London for monitoring and audit purposes.
5. I understand that confidentiality and anonymity will be maintained and it will not be possible to identify me in any research outputs.
6. I consent to any non-identifiable data being shared for research purposes with third parties which are within or outside the EU as outlined in the participant information sheet.
7. I agree to be contacted in the future by Addis Ababa University or King’s College London researchers who would like to invite me to participate in follow up studies to this project, or in future studies of a similar nature.
8. I agree that the research team may access my medical records for the purposes of this research project.
9. I agree that the research team may use my data for future research and understand that any such use of identifiable data would be reviewed and approved by a research ethics committee. (In such cases, as with this project, data would not be identifiable in any report).
10. I consent to selected sessions of problem-solving therapy (PST) or PST adapted to address IPV being audio recorded, if requested, for the purpose of quality monitoring.
11. I agree that my healthcare provider may be contacted if concerns about my mental or physical health or wellbeing are raised by this study, after discussion with me.
12. I understand that if I have any questions I can contact the study researchers.

**Statement of Consent**

I have read the participant information sheet or had it read for me. All my questions have been answered. I have read or been told about the purpose and safety of the study, what will be done and the risks and benefits of the study. I agree to be in the study.

**__________________ __________________ _________________**

**Name of Research Staff Date Signature**

**__________________ __________________ _________________**

**Name of Participant Date Signature (thumbprint)***

* In case the participant is not able to read this form or sign their name, this attests that the consent form has been read and explained accurately by a member of research staff in the presence of a witness, and that the participant has affixed their thumbprint as consent.

**Statement of a witness**

I, ________________________________ agree that the research project named above has been explained to ____________________________________(participant) to his/her satisfaction and that he/she agrees to take part in the study. Both the notes written above and the Information Sheet about the project have been read to him/her.

**__________________ _________________**

**Date Signature**

**Supplementary File 6c: Health Worker Information Sheet**

**INFORMATION SHEET FOR PARTICIPANTS**

*
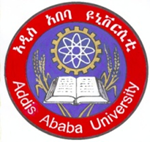
*
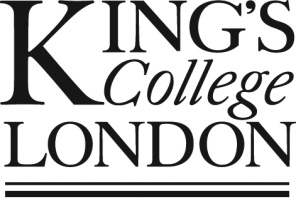


*KCL REC Ethical Clearance Reference Number:* HR-18/19-9230

*AAU IRB Ethical Clearance Reference Number: 032/19/CDT*

**YOU WILL BE GIVEN A COPY OF THIS INFORMATION SHEET.**

**TO PRESERVE CONFIDENTIALITY WE ASK THAT IT BE KEPT IN OUR LOCKED RESEARCH OFFICE.**

**Title of study:** A feasibility study of problem-solving therapy for depression and intimate partner violence in Ethiopia: Evaluation.

We would like to invite you to take part in our research project. This study is a collaboration between researchers from Addis Ababa University (AAU), King’s College London (KCL) in the United Kingdom (UK), and University of Cape Town (UCT) in South Africa. You should only participate if you want to; choosing not to take part will not disadvantage you in any way. Before you decide whether you want to take part, it is important for you to understand why the research is being done and what your participation will involve. Please take time to read the following information carefully or have the information read out to you, and discuss it with others if you wish. Ask us if there is anything that is not clear or if you would like more information.

**What is the purpose of the study?**

Stress, distress, feeling sad and becoming depressed are common experiences, including in pregnancy. Many women also experience violence in their relationship with their husband or partner, which is called ‘intimate partner violence’ (IPV). We have adapted a short talking therapy for depression called problem-solving therapy (PST) for Ethiopia. The version of problem-solving therapy which has been adapted to address IPV is called PST-IPV. This study will compare three different options for pregnant women with depressive symptoms. The purpose of this study is to find out if problem-solving therapy, problem-solving therapy adapted to address IPV and information can be compared in a randomised research study and to explore how acceptable and possible it is to provide them in this part of Ethiopia.

**Why am I being invited to take part?**

You are being invited to take part in this study because you participated in the randomised feasibility study as a healthcare worker or participant. A small number of staff and women are being asked to have a single interview after the feasibility study ends, as part of the evaluation.

**Do I have to take part?**

Participation is voluntary: you do not have to take part in this study. You should read this information sheet and if you have any questions you should ask the research team. If you decide to take part, you will be free to withdraw from the study at any time without needing to give a reason. Taking part or not taking part in this study will not affect your healthcare in any way. If you are uncomfortable answering certain questions you can refuse or ask to stop a session or questionnaire at any time, without needing to give a reason.

**What will happen if I take part?**

If you agree to take part, you will speak to the research assistant in a private room in a place and time that suits you best. They will record information about you, such as your age and level of education before asking a series of questions about your views and experiences of the study.

With your permission we will audio-record the interview so that we make sure we do not miss anything you tell us. Using the audio record, we will write down everything that is discussed in the interview. Once we have completed the analysis we will destroy the audio record. We expect that the interview will take about one hour to complete.

**What are the possible risks of taking part and how will they be addressed?**

A possible risk of you taking part in this study could be that other people know you are in the study. To avoid this happening, we will therefore take great care to keep your taking part confidential, for example by keeping your copy of this information sheet in our locked research office and organising sessions in a private room in a place and time convenient to you. All of your study information will be recorded with an identification number instead of your name. Paper records will be stored in a secure filing cabinet in our locked research office which has restricted access and electronic records will be stored on a secure computers or private computer websites.

For some people, when they speak about stress, distress, or husbands or partners being violent this could make them fell upset. If you feel upset during or after the interview, you can stop the interview at any time and the researcher will discuss this with you and offer additional support if you need and want it.

We also take very seriously any risks that we become aware of during the course of the study. If whilst taking part, you identify risks to your or another person’s health or safety, the researcher will speak to you about how to respond and provide support to you or the person. If there is an imminent danger of serious harm to someone, the police may need to be informed, after discussing this with you.

**What are the possible benefits of taking part?**

A benefit of this study is that it will give you a chance to give your opinion about how this study and care for women who are pregnant or postnatal can be improved.

**Compensation**

You will be compensated for your time and in addition you will be reimbursed for any travel or other expenses which you incur by taking part in the study.

**What happens if I decide not to take part in this study?**

Your participation in this study is entirely voluntary. Choosing to take part or not to take part in this study will not disadvantage you in any way.

Even after the study has started, you are free to stop taking part at any time, without giving a reason.

You can withdraw your data from the study up until 31/03/2020, after which withdrawal of your data will no longer be possible due to analysis commencing. If you choose to withdraw from the study we will not retain the information you have given thus far except for demographic information, which we will retain in order to report basic features of participants withdrawing from and remaining in the study.

**Will my taking part be kept confidential?**

Your interview responses will not be identifiable. The fact that you have taken part will be kept confidential. Your responses to questions will be analysed with other participants’ and will not be traceable to you in any reports.

**Data handling**

Your data will be processed in accordance with Ethiopian data protection rules and the United Kingdom’s (UK) General Data Protection Regulation 2016 (GDPR), which exist to protect you and your data. This means that:

Any of your personal data that has been collected will be destroyed two years after data analysis is completed. Following completion of this research, electronic records which are not identifiable to you will be stored on a secure computer which is protected by a password. Anonymised data will be available to future researchers so that we can learn as much as possible, but this will not be identifiable as your information.

Amharic language records will be shared (with your consent) with a transcription and translation service. Any potentially identifiable references made in translated records will be changed to preserve your confidentiality.

As this study is conducted between Ethiopia and the UK, some information that you tell us will be securely transferred between these countries (with your consent). However, this information will not be shared with any parties outside KCL, UCT and AAU. If any data need to be shared for research purposes, a special data transfer agreement will be in place to ensure that data continues to be held in compliance with Ethiopian data protection rules and UK data protection standards (GDPR).

**Data protection statement**

The data controller for this project will be KCL, who will process your personal data for the research described here. The legal basis for processing your personal data for research purposes under GDPR is a ‘task in the public interest’. You can give your consent for your personal data to be used in this study by completing the consent form we have provided.

You have the right to access information held about you. To discuss any of these rights or to exercise them, please inform the research assistant. Your right of access can be exercised in accordance with the General Data Protection Regulation.

**Who has reviewed this study?**

The ethics protocol of this study has been reviewed and approved by the Institutional Review Board of the College of Health Sciences of Addis Ababa University (AAU) and by the Psychiatry, Nursing and Midwifery (PNM) Research Ethics Subcommittee (RESC) at King's College London.

**How is the project being funded?**

This research is funded by King’s College London in the UK.

**What will happen to the results of the study?**

Based on your and other participants’ responses to this study, we will improve PST-IPV and the design of this study, to plan a larger study of problem-solving therapy adapted to address intimate partner violence and problem-solving therapy in Ethiopia. The information collected will enable us to design treatments that can be practically provided in this setting in a way that women and healthcare workers find helpful and acceptable.

We will present our findings to other researchers and they will be written about in a PhD thesis and peer-reviewed journal publications. You can request to receive a copy of the English language research from the study researchers (see below). All results will be presented without giving any personal details about you, so other researchers will not be able to identify you.

**What if I have further questions, or if something goes wrong?**

If you would like to talk to someone else about this project, if this study harmed you in any way or if you wish to make a complaint about the conduct of the study, you can contact the Institutional Review Board of the College of Health Sciences of Addis Ababa University, on 0115-5538734 or King's College London: The Chair, Psychiatry, Nursing and Midwifery (PNM) Research Ethics Subcommittee (RESC): [rec@kcl.ac.uk](mailto:rec@kcl.ac.uk).

**If I have some more questions about the research, who can I speak to?**

Please contact Research Assistant Adiyam Mulushoa, telephone number 0910 024757, at the College of Health Sciences, Addis Ababa University, Ethiopia. You can also contact Dr Tesera Bitew on 0911 173656.

**Thank you for reading this information sheet and for considering taking part in this research.**

**Supplementary File 6c: Health Worker Consent Form**

**CONSENT FORM FOR PARTICIPANTS IN RESEARCH STUDIES**

Please complete this form after you have read the Information Sheet and/or listened to an explanation about the research.

*
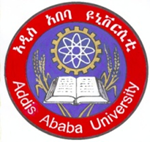
*
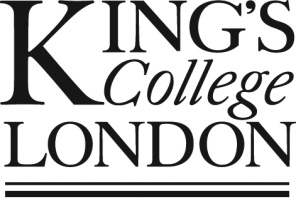


**Title of Study:** A feasibility study of problem-solving therapy for depression and intimate partner violence in Ethiopia: Evaluation

**KCL REC Ethical Clearance Reference Number:** HR-18/19-9230

**AAU IRB Ethical Clearance Reference Number:** 032/19/CDT

Thank you for considering taking part in this research. The person organising the research must explain the project to you before you agree to take part. If you have any questions arising from the Information Sheet or explanation already given to you, please ask the researcher before you decide whether to join in. You will be given a copy of this Consent Form to refer to at any time. To preserve your confidentiality, we ask that it be kept in our locked research office.

**Please tick or initial**

**I confirm that I understand that by ticking/initialling each box I am consenting to this element of the study. I understand that it will be assumed that unticked/initialled boxes mean that I DO NOT consent to that part of the study. I understand that by not giving consent for any one element I may be deemed ineligible for the study.**

**Please tick or initial**

1. I confirm that I have read and understood the information sheet dated 08.01.19, Version 1, for the above study. I have had the opportunity to consider the information and asked questions which have been answered to my satisfaction.
2. I consent voluntarily to be a participant in this study and understand that I can refuse to answer questions and I can withdraw from the study at any time, without having to give a reason, up until 31/03/2020.
3. I consent to the processing of my personal information for the purposes explained to me in the information sheet. I understand that such information will be handled in accordance with the terms of the General Data Protection Regulation and Ethiopian data protection rules.
4. I understand that my information may be subject to review by responsible individuals from Addis Ababa University and King’s College London for monitoring and audit purposes.
5. I understand that confidentiality and anonymity will be maintained and it will not be possible to identify me in any research outputs.
6. I consent to any non-identifiable data being shared for research purposes with third parties which are within or outside the EU as outlined in the participant information sheet.
7. I agree to be contacted in the future by Addis Ababa University or King’s College London researchers who would like to invite me to participate in follow up studies to this project, or in future studies of a similar nature.
8. I consent to my interview being audio recorded.
9. I agree that the research team may use my data for future research and understand that any such use of identifiable data would be reviewed and approved by a research ethics committee. (In such cases, as with this project, data would not be identifiable in any report).
10. I understand that if I have any questions I can contact the study researchers.

**Statement of Consent**

I have read the participant information sheet or had it read for me. All my questions have been answered. I have read or been told about the purpose and safety of the study, what will be done and the risks and benefits of the study. I agree to be in the study.

**__________________ __________________ _________________**

**Name of Research Staff Date Signature**

**__________________ __________________ _________________**

**Name of Participant Date Signature (thumbprint)***

* In case the participant is not able to read this form or sign their name, this attests that the consent form has been read and explained accurately by a member of research staff in the presence of a witness, and that the participant has affixed their thumbprint as consent.

**Statement of a witness**

I, ________________________________ agree that the research project named above has been explained to ____________________________________(participant) to his/her satisfaction and that he/she agrees to take part in the study. Both the notes written above and the Information Sheet about the project have been read to him/her.

**__________________ _________________**

**Date Signature**
